# Nature-Inspired Antimicrobial Surfaces and Their Potential Applications in Food Industries

**DOI:** 10.3390/foods11060844

**Published:** 2022-03-16

**Authors:** Aswathi Soni, Gale Brightwell

**Affiliations:** 1 Food Assurance, AgResearch, Palmerston North 4442, New Zealand; gale.brightwell@agresearch.co.nz; 2 New Zealand Food Safety Science Research Centre, Palmerston North 4442, New Zealand

**Keywords:** antimicrobial, natural surfaces, biofilms, AMR, food processing

## Abstract

Antimicrobial resistance (AMR) is a growing global concern and has called for the integration of different areas of expertise for designing robust solutions. One such approach is the development of antimicrobial surfaces to combat the emerging resistance in microbes against drugs and disinfectants. This review is a compressive summary of the work done in the field of material science, chemistry, and microbiology in the development of antimicrobial materials and surfaces that are inspired by examples in nature. The focus includes examples of natural antimicrobial surfaces, such as cicada wings or nanopillars, dragonfly wings, shrimp shells, taro leaves, lotus leaves, sharkskin, gecko skin, and butterfly wings, along with their mechanism of action. Techniques, compositions, and combinations that have been developed to synthetically mimic these surfaces against bacterial/viral and fungal growth in food-processing areas have also been discussed. The applications of synthetic mimics of natural antimicrobial surfaces in food-processing environments is still a naïve area of research. However, this review highlights the potential applications of natural antimicrobial surfaces in the food-processing environment as well as outlines the challenges that need mitigations.

## 1. Introduction

Antimicrobial activity can be explained as a property of a compound or structure that enables either inhibition, reduction in growth rate, or inactivation (cell death) of microbes. Antibiotic drugs have been reported in the literature since the discovery by Alexander Fleming of the first antibiotic, penicillin, in 1928 [1,2]. However, the use of plant extracts to treat infections by some of the earliest civilisations indicates their long existence. Despite the discovery of several antibiotics, a challenge that has simultaneously emerged in the last two decades is antibiotic resistance. Antibiotic resistance is also an emerging issue for food industries, as there is the pressure exerted by the primary sector in the use of antibiotics and biocides (e.g., disinfectants, food and feed preservatives, or decontaminants), which has led to an increase in the emergence of antimicrobial resistance throughout the food chain [3]. Another related concern is the ability of the resistant populations to form biofilms in food-processing areas. This includes biofilms formed by the spore-forming bacteria, which are very resistant to disinfectants and mild to moderate thermal wash [4], and biofilms from non-spore-forming bacteria, which eventually develop resistance to the treatment and can lead to severe food poisoning and spoilage issues, beyond their role in transmitting antimicrobial resistance (AMR) in the community [5]. Nevertheless, a limited number of disinfectants can be used within food-processing areas, as they might not only leave residues in the final products but can also damage the surfaces of equipment, making it further challenging to reduce bacterial contamination in the final product [4]. The regular use of disinfectants has also increased the selection of resistant bacteria, and this along with the resistance against antibacterial agents is an emerging challenge for the food-processing industries [6,7] and a serious concern to public health.

AMR, resistance to disinfectants, and persistence of biofilms in food-processing industries have urged researchers to search for alternative options against microbial contaminants. One such promising area is the investigation of surfaces/structures found in nature that exhibits an antimicrobial contact killing mechanism. This review comprises a summary of work reported in the last two decades on the discovery of natural and nature-inspired antimicrobial surfaces, techniques used in their development, and their potential applications in food industries against foodborne pathogens. At present, the research on antimicrobial surfaces is limited to either lab-scale or pilot-scale studies. This review summarizes the reports on the efficacy of antimicrobial surfaces on foodborne pathogens and spoilage-related microorganisms, which emphasises the future potential in the food sector.

## 2. Antimicrobial Surfaces in Nature

Antimicrobial surfaces consist of specialized surface architectures or chemical compositions, which either prevent the attachment of microbial species by limiting their adhesion or impart a bactericidal action by disrupting and damaging the cell organelles [8]. Some examples of natural antimicrobial surfaces and their activities reported against bacterial strains are summarized in Table 1. It is to be noted that the table only includes the reported studies on specific strains, and there is still a wide research gap on the evaluation of the efficacy of these surfaces onto other strains or on actual food-grade premises.

Insect wings (cicada wings, dragonfly wings, and butterfly wings) consist of nano protrusions on the surface, which are capable of damaging the bacterial cell membranes, thereby acting as an antibacterial surface [8,9,10]. These structures also limit the colonization of any surviving bacterial cells. The structure and pattern of the dragonfly wing have been well investigated by researchers. The nanostructures on the dragonfly wings can also be explained as nanopillars that are postulated to follow more than one type of model for their killing mechanism [17]. For example, as per the biophysical model, when a bacterial cell comes in contact with the cicada wing, nanopillars can physically stretch the cell membranes, which in turn leads to bacterial rupture and death [17]. Another model indicated a thermodynamic relationship between the bactericidal mechanism of nanopatterned surfaces and the total free energy change of bacterial cells in a patterned surface (like cicada wings), thereby leading to a drastic increase in the contact adhesion area [18]. The contact angle is an important attribute that plays a key role in the inhibition of bacterial attachment as well as in cell disruption [18].

Plant leaves have also been investigated for antimicrobial properties. Lotus leaf surfaces have been reported to demonstrate superhydrophobic properties due to the presence of an air cushion that is entrapped at the liquid/solid interfaces, which limits the contact area and minimizes the biofouling [19,20]. Additionally, lotus leaves have recently been reported to demonstrate bactericidal activity due to the presence of micro-sized papillae and nano-sized wax tubes on the outermost layer with a similar aspect ratio to the bactericidal nanopillars [19]. Cellulose nanofibers (8–10 nm in diameter) in plants, which are made up of β 1,4 linked glucopyranose units, are known to exhibit antibacterial activities [21]. A study by Bixler et al. [16] studied and reported the liquid–solid–air interface between rice leaf and the environment, which indicated that surface roughness and chemistry influences wettability since the air pocket encourages a larger contact angle, thereby preventing a great deal of deposition. A similar mechanism could be postulated to prevent the colonization of microbes on these surfaces.

Another example of a different antimicrobial surface is sharkskin, which has also been extensively studied and mimicked using additional metal implants for increasing the antifouling effect. The structure of the skin comprises small denticles that are flexed against each other due to the elasticity of the skin underneath [22]. The overall structure of the scales on sharkskin represents a repetitive pattern of parallel ridges and grooves, which are found to be placed at a distance of a few millimetres [22]. This complicated, irregular structure plays an important role in preventing microbial settlement and colonization of the surface [23,24]. Gecko skin also has unique, dome-shaped, and closely layered scales (diameter of 100–190 µm and height of 50 µm) arranged in a hexagonal pattern with the regular presence of spinules (tiny hairs) [15]. The exposure of bacterial cells to gecko skin has been reported to result in immediate destruction and therefore cell death due to possible cell damage by the spinules [15].

## 3. Mimicking Antimicrobial Structures/Materials

The knowledge acquired from natural antimicrobial surfaces has been now combined with nanotechnology-based design and engineering solutions to generate surfaces that can further enhance the utility and life of antimicrobial surfaces. Specific elements and compounds of interest for fabrication to mimic the antibacterial structures are listed in Table 2.

Synthetic black silicon is known to have high aspect ratios with unique surface properties, such as high hydrophobicity and strong biological activity [25,35]. The study by Ivanova et al. [25] demonstrated a similarity between the bactericidal action of black silicon and dragonfly wings and demonstrated a killing rate of 450,000 bacterial cells min^−1^·cm^−2^, indicating potential biomedical and industrial applications. Reactive ion beam etching was used in this study to produce silicon nanofibers to mimic the dragonfly wings. Gold sputter-coated wings of dragonflies (common sand dragon) have also been reported to exhibit similar lethal effects on fungal cells, especially against *Candida* isolates [8,36]. Although the fungal cell structure is very different to that of bacterial cells [37], it can be concluded that the nanoscale topography effects can be extended to fungal de-contamination. Inspired by the lotus leaf, mesoporous silica micro-patterns with a layering of 1 μm silicon oxide have been tested in developing the nanopillars [38]. A study by Green et al. [39] tested the bactericidal efficacy of synthetically replicated spinule arrays consisting of small nano-tipped hairs (spinules) inspired by gecko skin. Eight strains of *Lactobacillus* spp. (probiotic human bacteria strains) were used to study their ability to colonize on synthetically replicated spinule arrays. Although bacterial inactivation was not quantified, the results indicated a 95% reduction in the population due to a combination of stretching between spinules and nanotip piercing as monitored using scanning electron microscopy [39].

Several synergistic combinations of the compounds/elements mentioned in Table 2 are available in the literature. However, only a few of them, such as graphene and black silicon, has been extensively used in the development of fabricated antibacterial surfaces against foodborne pathogens, indicating a potential for new combinations.

### Advantages and Shortcomings of Nature-Inspired Antimicrobial Surfaces

Nanoengineered versions of the nature-inspired surfaces have their limitations as well as advantages as with any other technique/method that is currently in use. Some advantages include the physio-antimicrobial structure that does not rely on chemical components, which reduces the risk of developing chemical resistance or disinfectant-based chemical accumulation in the food chain [40]. Resistance to antibiotics and disinfectants is an emerging concern in the world, and the progress in the field of antimicrobial surfaces shows a potential step away from these chemical disinfectants. However, the use of certain chemicals in the formulation during nanoengineering leads to some limitations. For example, surfaces containing nano-silver, photocatalytic titanium oxides, and zinc oxide nanoparticles cannot be used for fresh produce disinfection or as an aerosol treatment for airborne disinfection, as they pose a risk of ingestion and inhalation [41]. Development of new antimicrobial surface and incorporation of antimicrobial polymers could be a lengthy and expensive process, and the financial gains could be long-standing benefits [42]. However, it can be postulated that with further research and progress on the development of nature-inspired, food-grade antimicrobial surfaces, the potential benefits, such as minimum biofilm formation, the longer shelf life of export quality products, and prevention of food recalls due to material-based cross-contamination, will eventually overcome the cost-associated challenges.

## 4. Effect of Antimicrobial Structures on Biofilms

Biofilms can be defined as communities of microorganisms that are attached to a surface with the help of polysaccharides and biomolecules secreted by them, and these groups act as a collective ecosystem consisting of one or many types of microbial contaminants that may be synergistic in nature [43,44]. Biofilm formation has been explained as a dynamic process that has five main steps: initial attachment, irreversible attachment, early development of biofilm architecture, maturation, and dispersion [4]. The freely suspended counterparts of the same organisms are known as planktonic cells, which differ significantly in their resistance, growth, and structure as compared to the cells/spores in biofilms [45]. Biofilms can be found on several types of surfaces, such as natural living tissues, natural aquatic systems, instruments of medical importance and functions, industrial machines, pipelines, drainage or potable water system piping, or even kitchen-based utensils. The factors influencing biofilm formation include secretion of extracellular matrix (ECM) by the individual planktonic cells. ECM consist of adhesins, polysaccharides, proteins, and DNA [46]. ECM has multiple functions in the sustainability of biofilms. This includes providing a glue-like medium for adhesion, protection against external stresses, and conferring a stable structure [47]. One of the key requisites in the formation of biofilms is the solid–liquid interface that needs to provide an optimum habitat for the cells to attach and grow. This can further be influenced by several factors, such as substratum, presence of other conditioning films on the substratum, hydrodynamics of the aqueous medium, characteristics of the medium, and the microbial strains involved [45,48]. Biofilms are a significant food safety concern for the food industries, as the strategies involved in cleaning and eradication are cost intensive, time-consuming, and in some cases might lead to huge disruptions in the processing lines and complete replacement of pipelines [49]. This can be further supported by reports demonstrating high acquired resistance by the cells in the biofilms when compared to their planktonic forms [50,51]. For example, a regular cleaning-in-place (CIP) regime used for planktonic cells that involve thermal treatment at 125 °C for 30 min could not lead to the complete inactivation of *Bacillus* spores in a 3-day-old biofilm [52]. A few examples of biofilm-forming bacteria and their relevance to food industries are indicated in Table 3.

The mechanism of action of natural antimicrobial surfaces against biofilms can be explained using two theories. As per the first one, natural antimicrobial surfaces can prevent biofilm formation by inhibiting the first step, i.e., the attachment of bacterial cells onto the target surface [8]. When the cells attach to the nanostructured surfaces of dragonfly wings, it is postulated to strongly adhere to the nanopillars using the extracellular polysaccharide (EPS) layer, which is then followed by shearing and membrane damage as the cell attempts to move across the nanotextured surface while it is immobilized [10].

Another theory based on the study by Linklater et al. (2017) proposed a different mechanism and indicated that the nanopillars might not be uniform in size in the *Orthetrum villosovittatum* dragonfly, which led to a different mechanism of cell damage unlike mechanistic models (using cicada wing nanopillars) [69]. The natural effect of nano topography of *O. villosovittatum* dragonfly wing on *E. coli* cells (involved in the formation of biofilm) indicated that although there is an initial attachment, the nanostructures lead to cell damage, which reduces proliferation and further sustainability of the biofilm [69]. The transmission electron micrographs indicated that the bacteria are attached to nanopillars via secreted EPS, with no direct contact of the membrane with the nanopillars. There is a large surface area attributed to the arrangement of nanopillars, which in turn leads to the initial adhesion through the EPS, followed by some movement of the bacterium. The attachment bends the taller nanopillars, which then penetrate the bacterial membrane, thereby rupturing the inner-cell membrane and the outer-cell membrane.

Carbon-infiltrated carbon nanotube (CICNT) surfaces have been reported to mimic a similar nanopillar structure to cicada wings, therefore conferring resistance to bacterial proliferation and biofilm formation by *Staphylococcus aureus* [70]. The mechanism of action is postulated to be the combined effect of hydrophobicity and size, as a result of which the bacterial cells do not get enough surface affinity for adhesion. A study by Cai et al. [71] investigated the effect of gecko cathelicidin on biofilm formation of *Streptococcus mutans* and reported interference in bacterial adhesion and the biofilm maturation stages. Cathelicidins are multifunctional antimicrobial peptides, which are effective against EPS synthesis, acid production, and bacterial acid tolerance. Although *S. mutans* are more relevant in the field of dentistry due to their carcinogenic potential and resistance to disinfection, the findings indicate a potential against foodborne pathogens especially, as this study emphasized low cytotoxicity and haemolysis of cathelicidin (Gj-CATH2) on mammalian cells [71]. A study by Chein et al. [72] investigated the role of the microscale structure of sharkskin on early bacterial attachment and biofilm formation by *S. aureus* and *E. coli.* This study evaluated the biofilm formation for 14 days, and it was evident that in comparison to any flat surface, the sharkskin (natural and synthetic) led to a significantly lesser number of viable cells in the biofilm. This also indicated that there was no significant difference in the attachment (measured using absorbance values); however, the growth, proliferation, and development of biofilm was reduced on sharkskin as compared to a flat surface that was used as a control [72]. Sharkskin consists of riblet ridges along the body axis, which act as a barrier to the flow of water close to the skin and potentially reduce the drag on the body. This, in turn, inhibits bacterial attachment and growth and hence acts as an antifouling agent. The epidermal mucus (consisting of antimicrobial peptides) of sharkskin inhibits the growth of a variety of microbes, and the structural micrography also reduces the settlement of bacterial contaminants; all of these factors are postulated to help against biofilm formation [72,73,74]. Synthetic nanofibrils of polypropylene (PP) that mimic the structure of the lotus leaf, consisting of low surface energy and hierarchical microstructure, were reported to be effective in reducing biofilm formation by *E. coli* [75]. The hydrophilic surface of PP was created using a technique known as oxygen and fluorine reactive ion etching (RIE), which led to changes in wettability and therefore reduced the bacterial adhesion by 68.7% as compared to untreated PP [75]. The superhydrophobic nature of taro (*Colocasia esculenta*) leaves has also been known to confer antifouling properties against bacteria [12]. The hydrophobic epicuticle layer on taro leaves consists of nanosized wax crystalloids that are convex, micro-sized surface structures, which create a high contact angle [12]. As a result, microbial cells fail to attach to the surface, and therefore, biofilm formation is inhibited. Taro leaves have been reported to show prevention of adhesion against *Pseudomonas aeruginosa* under both wet and dry conditions [12], indicating potential application in processing areas where the moisture content is high.

These findings indicate a significant potential of the antimicrobial surface topographies and surface designs that can be used in food-processing areas, which would increase the life of structures that are often compromised due to biofilms. The efficacy is also postulated to be increased by using synthetic bactericidal compounds/elements listed in Table 2.

### 4.1. Novel Techniques Used for the Development of Nanostructures

Antimicrobial coatings to enhance the antimicrobial efficacy of materials that are used to design and engineer surfaces that are based on learnings from nature have two main objectives: (1) controlled release of antibiotic agents and (2) eliminating free nanoparticles. Some examples of nanocoating are nanocomposite films containing metal nanoparticles, silver nanoparticles, copper nanoparticles, and zinc oxide nanoparticles. The traditional “wet” process of nanocoating involves the use of physical vapour deposition (PVD) or chemical vapour deposition (CVD). However, new techniques have been regularly sought and reported. Some novel techniques have been widely used to modify surfaces and have the advantage of being suitable for a wide range of polymers, ceramics, and metal surfaces. With this method, there are various models of protocols proposed. For example, the surfaces are first exposed to plasma for surface activation and then immersed into an aqueous antimicrobial component solution for absorption. Silver plasma immersion ion implantation process has been reported to be effective in grafting silver nanoparticles on titanium substrates, which were effective in inhibiting the growth of *E. coli* and *S. aureus* cells [76]. This technology has also been useful in improving the efficacy of bacterial inhibition on silver-treated polyurethane, which is the material used to manufacture catheters [77]. In a study by Gray et al. [77], the process involved silver deposition on the polymer films by a conventional electroless plating technique, followed by plasma treatments using a 2.45 GHz microwave generator. The growth inhibition of *E. coli* significantly improved polyurethane surfaces, and an inverse relationship between silver coverage and antibacterial efficacy was observed due to a reduced dissolution rate, with increasing particle size leading to less efficacy [77]. Another potential technique of ion deposition [78,79], especially using silver ion coating on stainless steel surfaces, has shown broad-spectrum antibacterial activities with potential applications in food industries [79].

Polymer-based nanocomposite coatings (PBNCs) consist of two phases; one is the matrix made up of polymers, and the other is nanoparticles. Antimicrobial polymeric nanomaterials have also gained a great deal of attention in this field due to their antibacterial/bactericidal action via different mechanism actions. For example, polymer-coated titania nanoparticles have demonstrated photocatalytic antibacterial properties as demonstrated by inactivation efficiency (95.7%) against *S. aureus*, which was further enhanced in the presence of UV (312 nm) light [80]. The mechanism of action was postulated as photogenerated reactive oxygen radicals that attack and disrupt the bacterial cell wall, resulting in cell death [80]. Silver nanoparticle fabricated into chitosan using stainless steel has been reported to be efficient against *S. aureus* as tested using disc diffusion-based assay for the zone of inhibition [81]. The mechanism of action is attributed to the slow release of silver ions from the polymer matrix, which is known to exhibit bactericidal properties due to either inhibition of the respiratory enzymes, disruption of metabolic activities, or the nucleic acid damage leading to consequent death of the microorganism [81,82]. Polymer (poly(N-isopropyl acrylamide))-coated silica nanoparticles have also demonstrated efficacy against *Bacillus* species (vegetative cells) as assessed using a preliminary growth inhibition assay via disc diffusion [83]. However, quantification of reduction in bacterial population was not conducted. Further developments and research on the biocompatibility of various materials used for antimicrobial grafting and coating along with the stability of these structures over time would increase applications in the processing industries.

### 4.2. Bioinspired Antimicrobial Peptides and Their Applications on Antimicrobial Surfaces

Antimicrobial peptides are naturally occurring antimicrobial peptides, which are cationic molecules that show resistance and inhibition against bacteria, some viruses, fungi, and parasites [84]. A few examples of antimicrobial peptides, which could be developed as potential alternatives to antibiotics, are summarised in Table 4. The mechanism of action by these antimicrobial peptides is through disruption of plasma cell membranes of the bacterial targets. The antimicrobial action is influenced by amphipathicity, hydrophobicity, structural folding, and polarity [85] depending on the structural specialities of these peptides. Researchers have been successful in generating synthetic antimicrobial peptides (SAMPs) that mimic the confirmation and activity up to various extents. Some examples are listed in Table 4.

Antimicrobial peptides derived from humans, plants, animals, and bacteria are under research, which aims to understand the opportunities of grafting them on the surface of polymers to thereby increase the antimicrobial activity [95]. This application would have two benefits; the natural physical topography of the surfaces will prevent biofilm formations, and the leaching ability of the antimicrobial peptides will add another layer of protection. However, the success largely depends upon the stability of the chemical bond established between the polymers and the AMPs. Grafting or immobilization of the AMPs on the polymer surfaces is accomplished using either physical or chemical methods. Examples of physical methods used in this regard are adsorption, layer-by-layer (LbL), and chemical methods (covalent bonding and Self-Assembled Monolayers (SAMs)) [96,97,98,99]. One of the challenges yet to be addressed is to develop methods to ensure the controlled release of grafted peptides to an extent that a minimum inhibitory concentration for commencement of antibacterial mechanism of action [100]. For the antibacterial action of these AMPs, the minimum concentration needs to be leached out, which can then disrupt the bacterial cell membranes by the formation of pores, followed by extended damage to the organelles, which lead to cell death [101,102]. Although further research in this field is required to facilitate the rational design of novel antimicrobial agents, it offers a great potential to be incorporated into surfaces for use in food-processing areas. Natural antimicrobial peptides (with no synthetic derivatives) that are extracted from microbes, plants, and animals have been well investigated and reported in literature and hence have not been included in this review.

## 5. Challenges and Research Gap

While antibiotic resistance has been widely reported in the literature, another challenge that has attracted food-safety experts is the emerging resistance to sanitization (antiseptics and disinfectants) [103,104]. The mechanism of resistance to sanitisers by bacterial cells could be (not limited to) due to reduced cellular permeability (gram-negative bacteria and spores), genetic mutations leading to acquired cellular mutations [105], and plasmid/transposon-mediated resistance [106]. Food-borne outbreaks due to consistent persistence of the pathogens in food-handling areas would continue with resistance to disinfectants, thereby posing a threat to human health. However, due to the limited research on specific disinfectants and target strains, information on mutational changes leading to resistance and the factors influencing these changes remain elusive. Nevertheless, the deposition of compounds/elements from disinfectants in the environment can attribute to the co-selection of multidrug-resistant bacteria [107,108,109]. Therefore, the development of alternative strategies, such as antimicrobial surfaces to reduce the use of disinfectants, is necessary. One of the most exciting future applications of nature-inspired novel antimicrobial structures is their use in inhibiting and inactivating AMR and sanitiser-resistant strains, which is a long-term concern across the globe and offers an alternative strategy for the management of foodborne illness.

**Figure 1:**
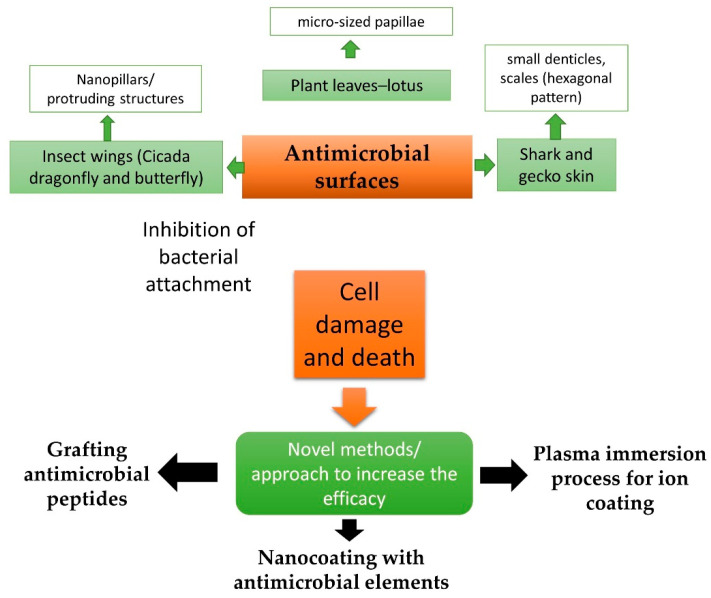
A summary of the progress on antimicrobial surfaces.

A summary of the work presented in this review is shown in Figure 1.

The antimicrobial mechanisms of the naturally occurring surfaces, such as insect leaves, skins, and leaf surfaces, are known to be either physical or mechanical in origin. However, there is an unexplored plethora of information on how the cell membranes and structures of various microbial cells could be affected by these. For example, the theory behind the inhibition of biofilms covers two aspects; Bandara et al. [10] indicated inhibition of attachment, while Linklater et al. (2017) suggested that proliferation of cells in biofilms is prevented [69]. Similarly, less information exists on the antimicrobial effects of the nanofibrils or topographic morphologies on bacterial spore formers, such as *Bacillus* and *Clostridium* species. The spores form an extensively resistant external cortex that prevents them from external stresses, such as desiccation, pH, or even chemical disinfectants. The effect of nanostructures on bacterial spores remains elusive. Thermal treatment and high-pressure processing have been reported as effective ways to inactivate spores in food [110,111,112]. The removal of spores from the surfaces heavily relies on application-specific disinfectants [113]. Therefore, antimicrobial surfaces effective against spores would be beneficial for food industries.

Another research gap includes the specific effect on biofilms that comprise both vegetative cells and spores, such as *B. cereus*. While the efficiency against biofilms consisting of vegetative cells has been partially reported and highlighted in this review, the spores in biofilms are known to be more resistant to CIP regimes. The use of novel techniques, such as plasma activation and ion deposition, to improve the efficiency of antimicrobial surfaces indicates potential. However, the possibility to use them in food-grade premises is yet to be investigated, especially due to the long- and short-lived activated ions, which might affect the environment and food. The long-term efficacy of the antimicrobial surfaces, especially in the presence of regular wear and tear in a processing environment, is yet to be investigated, which would strengthen the prospects of applications. Additionally, many antibacterial surfaces are effective only in the presence of an aqueous solution and may prove less effective in killing airborne bacteria in the absence of a liquid medium [114]. Studies on the effect of these surfaces on the attachment of foodborne viruses, such as noroviruses, hepatitis A and E viruses, rotaviruses, and astroviruses [115], are not available in the literature. While viruses need a host cell to replicate and infect, the non-living surfaces can act as a carrier or transport between the host and viruses, and therefore, the inhibition of the initial adhesion of viruses would reduce the transmission.

AMR has now been reported to be associated with foodborne pathogens due to the use of antimicrobials in both animals and humans, which is postulated to select for resistant bacterial populations [116]. There is an evident lack of data on the efficacy of antibacterial surfaces and their mechanism of action against the major AMR strains, such as *Klebsiella pneumoniae*, non-typhoidal and typhoidal *Salmonella* and *Mycobacterium tuberculosis*, *Campylobacter jejuni*, *L. monocytogenes*, and *Yersinia enterocolitica.*

## 6. Conclusions and Future Perspective

This review summarizes the currently available studies that investigated the application of nature-inspired antimicrobial surfaces in the inhibition of foodborne pathogens and biofilm formation. Food-processing industries face a challenge of limited availability in the number of disinfectants, which could be used in food-grade environments. The increase in multidrug-resistant bacterial strains as well emerging resistance against disinfectants warrants alternative strategies that could use physical topologies to inactivate bacteria or reduce their proliferation. The provision of antimicrobial nature-inspired surfaces, in a combination of nano-particles, could be used for the fabrication of food-grade surfaces, especially in hard-to-reach areas, which might significantly reduce the requirement of chemical use in processing areas. This would not only be economical but also environmentally sustainable due to the minimum release of chemicals in the food chain. Further research is required to investigate the mechanisms of microbial inactivation, suitable materials and surface structure, as well as the hardiness and safety of these surfaces in food-processing environments.

Future research should consist of studies that evaluate the shelf life, efficacy, and economic perspectives of using nature-inspired nanomaterials in the food industries. The incorporation specifically should investigate the inhibition of biofilm formation by foodborne pathogens that are resistant to antibiotics and disinfectants. The studies (highlighted) in this review indicate the potential of using nanostructures in combination with techniques such as plasma immersion and ion etching, which can further enhance the activity of these structures. The incorporation of nanostructures in hard-to-reach kitchen areas in restaurants would be the stretch-goal using these structures; however, this would involve accomplishment of economic feasibility. Studies that validate the broad-spectrum efficacies of antimicrobial surfaces against bacteria, viruses, and fungi would support the applications of the next generation of antimicrobial surfaces in food industries.

## Figures and Tables

**Table 1:** List of natural antimicrobial structures.

Antimicrobial Surfaces	Microbial Strains Tested	References
Cicada wings—Nanopillars	*Staphylococcus aureus*, *Escherichia coli,* and *Klebsiella pneumoniae*	[9]
Dragonfly wings	*Escherichia coli*	[10]
Chitosan from shrimp shell	*Escherichia coli* and *Staphylococcus aureus*	[11]
Taro leaves	*Pseudomonas aeruginosa*	[12]
Lotus leaves	*E. coli* and *S. aureus*	[13]
Sharkskin	*E. coli or S. aureus*	[14]
Gecko skin	*Porphyromonas gingivalis*	[15]
Butterfly wing	*Escherichia coli*	[16]

**Table 2:** Synthetic bactericidal compounds/elements used in developing antimicrobial surfaces.

Bactericidal Compounds/Elements	Unique Properties	Efficacy Study	References
Black silicon	High aspect ratios like that of a dragonfly wing.	Antibacterial against gram-negative and gram-positive bacteria and endospores	[25]
Graphene, graphene oxide (GO), reduced GO (rGO), and graphene quantum dots (GQDs)	Photo activator properties of graphite oxide	Efficiency against *S. aureus*, *E. coli, Pseudomonas aeruginosa*, and *Bacillus subtilis*	[26,27]
Titanium	Optical transparency and refractive index and wide bandgap energy, photocatalytic activity	Efficient against *E. coli* and *S. aureus*	[28,29,30]
Silver	Hydrophobic surface to inhibit the growth of bacterial flora	Efficient against *E. coli* and *B. subtilis*	[31,32]
Gold	Large surface-to-volume ratio	Efficient against *Salmonella typhimurium, Salmonella enteritidis, Salmonella typhi*	[33]
Zinc	Sulfhydryl reactivity of the ionic compound	Efficient *Streptococcus mutans*	[34]

**Table 3:** Biofilms in food industries—microbial strains of concern.

Biofilm-Forming Strains	Industrial Concern	References
*Bacillus cereus*	Negatively affects product quality and safety in dairy products. Produces emetic (cereulide) and enterotoxins (non-haemolytic enterotoxin, haemolysin BL, cytolysin K).	[53,54]
*Geobacillus stearothermophilus*	A common contaminant in powdered dairy products causing spoilage after reconstitution.	[55]
*Pseudomonas* spp.	Has been reported in dairy, meat, fresh produce, as well as ready-to-eat meal industries. Is capable of producing a high concentration of extracellular matrix (ECM) for strong attachment and is psychrophilic.	[56,57]
*Aeromonas hydrophila*	Has been reported in produce and seafood industries, leading to product contamination, food poisoning, and zoonotic diseases.	[58,59,60]
*Listeria monocytogenes*	Dairy, meat, fish, chilled vegetables, and ready-to-eat products have been known to be affected. Contamination leads to listeriosis outbreaks and therefore recalls.	[61,62,63,64]
*Escherichia coli*	Dairy, meat, seafood industries are commonly affected. Reduction of shelf life, a food poisoning outbreak, and recalls have been reported, especially with Shiga toxin-producing *E. coli* (STEC).	[65,66,67]
*Staphylococcus aureus*	Shelf-life reduction and food-safety concerns in meat-, poultry-, and dairy-processing industries.	[68]

**Table 4:** Bioinspired peptides against foodborne pathogens.

Natural Peptides	Bioinspired Derivatives Peptides	Bactericidal Effect against Strains	References
Magainins from the skin of the African frog *Xenopus laevis*	Disulphide-Dimerized Magainin Analogue	*Stenotrophomonas maltophilia* and *Escherichia coli.*	[86]
Cathelicidins in humans	SMAP-29, a cathelicidin-derived peptide from sheep myeloid mRNA	Potent antimicrobial activity against antibiotic-resistant clinical isolates of *S. aureus* (MRSA), vancomycin-resistant *Enterococcus faecium* (VREF), and mucoid *P. aeruginosa*	[87,88]
Defensins in humans	Ornithodoros defensin A	Bactericidal activity against *Bacillus cereus*, *Enterococcus faecalis*, and methicillin-resistant *Staphylococcus aureus*	[89]
Cationic peptides (1 and 2) derived from rabbit lung macrophages	Synthetic CAP18 (106–142)	Antibacterial effect on *Bacillus subtilis*, *Listeria monocytogenes*, and *Streptococcus faecalis*	[90,91,92]
Bactenecin-Innate defence regulator peptide-1018 (IDR-1018)	1018-derivative peptide named 1018-K6	Bactericidal efficiency specifically against *Listeria monocytogenes*	[93]
Cecropin A, the naturally occurring peptide in moths	CM15 synthetic peptide	Bactericidal effect on *Escherichia coli*	[94]

## Data Availability

Data is contained within the article.
